# Supplementary material for: Comparative multiomics analysis of cell physiological state after culture in a basket bioreactor
Source: Sci Rep. 2022 Nov 23;12:20161. doi: 10.1038/s41598-022-24687-4 (PMC9686226; doi:10.1038/s41598-022-24687-4)
Supplement: Supplementary file 1 — Supplementary Information 1. [file 41598_2022_24687_MOESM1_ESM.zip › raw data/Proteomic raw data/results_dir.html]

Directory Tree


# Directory Tree

../results
  
 ├── 0. raw.data: 原始数据 
  
 │   ├── 0.1. raw\_file: 质谱下机数据 
  
 │   └── 0.2. match\_result: 质谱搜库的结果文件 
  
 │
  
 ├── 1. data\_control: 数据质控结果 
  
 │
  
 ├── 2. func.anno: 蛋白功能注释结果 
  
 │   ├── 2.1. GO: GO功能注释结果 
  
 │   ├── 2.2. COG: COG功能注释结果 
  
 │   ├── 2.3. KEGG: KEGG功能注释结果 
  
 │   ├── 2.4. IPR: IPR（结构域）注释结果 
  
 │   └── 2.5. stat: 注释结果的整合 
  
 │
  
 ├── 3. ProtExprQuantification: 蛋白表达水平结果 
  
 │
  
 ├── 4. DiffExprAnalysis: 蛋白差异表达结果(定性项目无此内容) 
  
 │
  
 ├── 5. GOenrichment: GO富集结果(定性项目无此内容) 
  
 │
  
 ├── 6. KEGGenrichment: KEGG富集结果(定性项目无此内容) 
  
 │
  
 ├── 7. IPRenrichment: IPR富集结果(定性项目无此内容) 
  
 │
  
 ├── 8. Subcellular\_Diff: 差异蛋白亚细胞定位分析(定性项目无此内容) 
  
 │
  
 └── 9. PPI: 差异蛋白互作分析(定性项目无此内容)

---

tree v1.5.3 © 1996 - 2009 by Steve Baker and Thomas Moore   
HTML output hacked and copyleft © 1998 by Francesc Rocher   
Charsets / OS/2 support © 2001 by Kyosuke Tokoro
